# Supplementary material for: Empirical methods for controlling false positives and estimating confidence in ChIP-Seq peaks
Source: BMC Bioinformatics. 2008 Dec 5;9:523. doi: 10.1186/1471-2105-9-523 (PMC2628906; doi:10.1186/1471-2105-9-523)
Supplement: Additional File 1 — A variety of html documents from the USeq web site detailing the available applications, their best usage, output file type descriptions, command line menus, etc. [file 1471-2105-9-523-S1.zip › USeqUserGuides/JavaDoc.zip!/JavaDoc/trans/tpmap/IntensityFeature.html]

IntensityFeature


|  |  |  |
| --- | --- | --- |
| **Overview** \| **Package** \| **Class** \| **Use** \| **Tree** \| **Deprecated** \| **Index** \| **Help** | |  |
| **PREV CLASS**   **NEXT CLASS** | **FRAMES**    **NO FRAMES**     **All Classes** |  |
| SUMMARY: NESTED \| FIELD \| CONSTR \| METHOD | DETAIL: FIELD \| CONSTR \| METHOD |  |


---


## trans.tpmap Class IntensityFeature

```
java.lang.Object
  trans.tpmap.IntensityFeature
```

**All Implemented Interfaces:**: java.io.Serializable, java.lang.Comparable

---

``` public class IntensityFeature extends java.lang.Object implements java.lang.Comparable, java.io.Serializable ```

Minimal info about a feature for combining multiple chips together.

**See Also:**: Serialized Form

---

| **Field Summary** | |
| --- | --- |
| `float` | `intensity` |
| `static long` | `serialVersionUID` |
| `int` | `start` |


**Constructor Summary**

`IntensityFeature(MapFeature feature,
float intensity)`


| **Method Summary** | |
| --- | --- |
| `int` | `compareTo(java.lang.Object obj)`             Sorts by position. |

**Methods inherited from class java.lang.Object**

`equals, getClass, hashCode, notify, notifyAll, toString, wait, wait, wait`

**Field Detail**

### serialVersionUID

```
public static final long serialVersionUID
```

**See Also:**: Constant Field Values

---


### start

```
public int start
```

---


### intensity

```
public float intensity
```


**Constructor Detail**

### IntensityFeature

```
public IntensityFeature(MapFeature feature,
                        float intensity)
```


**Method Detail**

### compareTo

```
public int compareTo(java.lang.Object obj)
```

:   Sorts by position.

    :   **Specified by:**: `compareTo` in interface `java.lang.Comparable`


---


|  |  |  |
| --- | --- | --- |
| **Overview** \| **Package** \| **Class** \| **Use** \| **Tree** \| **Deprecated** \| **Index** \| **Help** | |  |
| **PREV CLASS**   **NEXT CLASS** | **FRAMES**    **NO FRAMES**     **All Classes** |  |
| SUMMARY: NESTED \| FIELD \| CONSTR \| METHOD | DETAIL: FIELD \| CONSTR \| METHOD |  |


---
